# Rapid chromosome territory relocation by nuclear motor activity in response to serum removal in primary human fibroblasts

**DOI:** 10.1186/gb-2010-11-1-r5

**Published:** 2010-01-13

**Authors:** Ishita S Mehta, Manelle Amira, Amanda J Harvey, Joanna M Bridger

**Affiliations:** 1 Centre for Cell and Chromosome Biology, Division of Biosciences, School of Health Sciences and Social Care, Brunel University, Kingston Lane, Uxbridge, UB8 3PH, UK; 2 Brunel Institute for Cancer Genetics and Pharmacogenomics, Division of Biosciences, School of Health Sciences and Social Care, Brunel University, Kingston Lane, Uxbridge, UB8 3PH, UK

## Abstract

**Background:**

Radial chromosome positioning in interphase nuclei is nonrandom and can alter according to developmental, differentiation, proliferation, or disease status. However, it is not yet clear when and how chromosome repositioning is elicited.

**Results:**

By investigating the positioning of all human chromosomes in primary fibroblasts that have left the proliferative cell cycle, we have demonstrated that in cells made quiescent by reversible growth arrest, chromosome positioning is altered considerably. We found that with the removal of serum from the culture medium, chromosome repositioning took less than 15 minutes, required energy and was inhibited by drugs affecting the polymerization of myosin and actin. We also observed that when cells became quiescent, the nuclear distribution of nuclear myosin 1β was dramatically different from that in proliferating cells. If we suppressed the expression of nuclear myosin 1β by using RNA-interference procedures, the movement of chromosomes after 15 minutes in low serum was inhibited. When high serum was restored to the serum-starved cultures, chromosome repositioning was evident only after 24 to 36 hours, and this coincided with a return to a proliferating distribution of nuclear myosin 1β.

**Conclusions:**

These findings demonstrate that genome organization in interphase nuclei is altered considerably when cells leave the proliferative cell cycle and that repositioning of chromosomes relies on efficient functioning of an active nuclear motor complex that contains nuclear myosin 1β.

## Abstract 2

Nuclear myosin 1β-dependent repositioning of chromosome territories occurs within 15 minutes of serum starvation in human cells.

## Background

Within interphase nuclei, individual chromosomes are organized within their own nuclear space, known as chromosome territories [1,2]. These interphase chromosome territories are organized in a nonrandom manner in the nuclei of human cells and cells from other species [3]. Chromosomes in different species are positioned radially, according to either their gene density [4-9] or their size [10-12] or both [11,13-16]. The nuclear microenvironment within which a chromosome is located could affect its gene regulation, and it has been proposed that whole chromosomes or regions of chromosomes are shifted around the nucleus to control gene expression [17,18]. Active genes appear to come together in a common nuclear space, possibly to be co-transcribed [19-21]. This fits with the increasing number of observations made of chromosome loops, containing active areas of the genome, coming away from the main body of the chromosome territory, such as regions containing *FLNA *on the X chromosome [22]; major histocompatibility complex (*MHC*) genes [23], specific genes on chromosome 11 [24]; β- globin-like genes [25], epidermal differentiation complex genes [26], specific genes within the *Hox B *cluster [27,28], and genes inducing porcine stem cell differentiation into adipocytes [29]. Chromatin looping is apparently associated with gene expression, because inhibition of RNA polymerase II transcription affects the outward movement of these chromosome loops [30].

Repositioning of whole chromosome territories has been observed in erythroid differentiation [25], adipogenesis [31], T-cell differentiation [32], porcine spermatogenesis [33], and after hormonal stimulus [34]. Even more studies revealed genomic loci being repositioned during differentiation (see [35], for comprehensive review). We demonstrated previously that interphase chromosomes occupy alternative nuclear positions when proliferating cells become quiescent or senescent [5,7,9]. For example, chromosomes 13 and 18 move from a peripheral nuclear location to an internal nuclear location in serum-starved or senescent fibroblast cells [5,9]. From these early studies, it was not clear how other chromosomes behaved after induction of growth arrest, and so we have now positioned all human chromosomes in cells made quiescent by serum starvation. We found that just less than half of the chromosomes alter their nuclear location. The ability to control, temporally, the entry of cells to quiescence through serum starvation allows the determination of a response time of nuclear architecture to the change in environment. In this study, we demonstrate that chromosome repositioning in interphase nuclei occurs within 15 minutes.

The presence of actin [36] and myosin [37-41] have been reported in nuclei, and an increasing body of evidence suggests that they cooperate to form a nuclear myosin-actin motor [42]. Actin and myosin have been shown to be involved in the intranuclear movement of chromosomal regions [43,44] and whole chromosomes [34]. Further, nuclear actin and myosin are involved in RNA polymerase I transcription [37,40], RNA polymerase II transcription [37-41], and RNA polymerase III transcription [45]. In a model put forward by Hoffman and colleagues [42], myosin I could bind through its tail to the nuclear entity that requires movement, with actin binding to the globular head of the nuclear myosin I molecule. This nuclear motor would then translocate the nuclear entity along highly dynamic tracks of nuclear actin [42]. In this study, we demonstrated that the rapid movement of chromosome territories in response to serum deprivation is dependent on the function of both actin and myosin, probably nuclear myosin 1β.

## Results

### Interphase chromosome positioning in proliferating and nonproliferating cells

**Figure 1:**
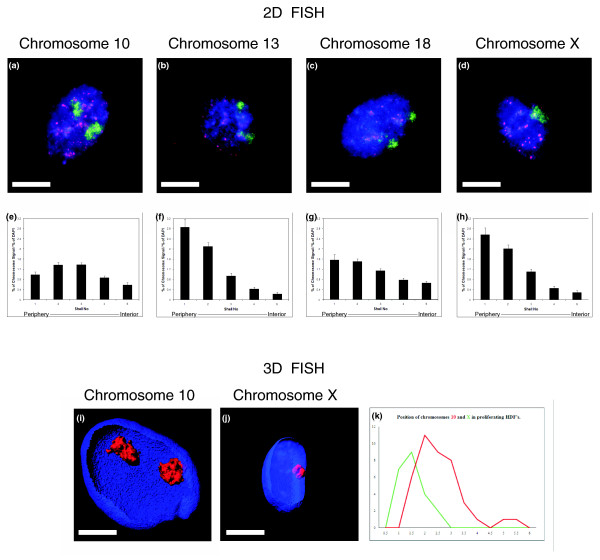
**Chromosome positioning in proliferating interphase nuclei**. Proliferating human dermal fibroblasts (HDFs) cultures were subjected to 2D- or 3D-fluorescence *in situ *hybridization (FISH) to delineate and analyze the nuclear location of chromosomes 10, 13, 18, and X. In panels **(a-d)**, the chromosome territories are revealed in green with a single chromosome territory for chromosome X, because the HDFs are male in origin. The red antibody staining is the nuclear distribution of the proliferative marker anti-pKi-67, the presence of which denotes a cell in the proliferative cell cycle. DAPI (4',6-diamidino-2-phenylindole) in blue is a DNA intercalator dye and reveals the nuclear DNA. Scale bar = 10 μm. The histograms in panels **(e-h) **display the distribution of the chromosome signal in 50 to 70 nuclei for each chromosome for 2D FISH, as analyzed with erosion analysis. This analysis divides each nucleus into five concentric shells of equal area, with shell 1 being the most peripheral shell, and shell 5 being the most interior shell [4-6,9]. The percentage of chromosome signal measured in each shell was divided by the percentage of DAPI signal in that shell. Bars represent the mean normalized proportion (percentage) of chromosome signal for each human chromosome. Error bars represent the standard error of the mean (SEM). Panels **i **and **j **display 3D projections of 0.2-μm optical sections through 3D preserved nuclei subjected to 3D-FISH and imaged with confocal laser scanning microscopy. The chromosome territories are displayed in red, and proliferating cells also were selected with positive anti-pKi-67 staining (not shown in reconstruction). Scale bar = 10 μm. The line graph in panel **(k) **displays a frequency distribution of micrometers from the geometric center of the chromosome territories to the nearest nuclear periphery, as defined by DAPI staining. Images for 20 nuclei were analyzed.

To determine the nuclear location of specific chromosomes, human dermal fibroblasts (HDFs) were harvested and fixed for standard 2D-fluorescence *in situ *hybridization (FISH). Representative images of chromosome territories in proliferating cells are displayed in Figure 1a-d. Digital images were subjected to erosion analysis [4-6,8,9], whereby the images of 4',6-diamidino-2-phenylindole (DAPI)-stained flattened nuclei are divided into five concentric shells of equal area, and the intensity of the DAPI signal and probe signal is measured in each shell. The chromosome signal is then normalized by dividing it by the percentage of DAPI signal. The data for each chromosome are then plotted as a histogram with error bars, with the x-axis displaying the nuclear shells from 1 to 5, representing the nuclear periphery to the nuclear interior, respectively (Figure 1e-h).

In young proliferating fibroblasts, interphase chromosomes are positioned nonrandomly in a radial pattern within nuclei [3]. In our 2D studies, we consistently found gene-poor chromosomes, such as chromosomes X, 13, and 18, located at the nuclear periphery [5,9], which fits with their having more lamina-associated domains than gene-poor chromosomes (see [46]). In this study, we recapitulated the interphase chromosome positioning with our present cultures and demonstrated that these chromosomes are located at the nuclear periphery in young proliferating cells (Figure 1b-d, f-h). Proliferating cells within the primary cultures were identified by using the proliferative marker, anti-pKi-67, which is distributed in a number of different patterns within proliferating human fibroblasts [47]. Its distribution is mainly nucleolar and is shown in red (Figure 1a-d). Figure 1a and e demonstrate the nuclear location of chromosome 10, unlike chromosomes 13, 18, and X it is found in an intermediate position in proliferating fibroblasts. The relative interphase positions of chromosomes 10 and X have been confirmed in 3D-FISH analyses (Figure 1i-k), whereby HDFs were fixed to preserve their three-dimensionality with 4% paraformaldehyde and subjected to 3D-FISH [48]. Measurements in micrometers from the geometric center the chromosome territories to the nearest nuclear periphery, as determined by the DAPI staining, were taken in at least 20 nuclei. The data were not normalized for size measurements, so that actual measurements in micrometers can be seen. However, all data were normalized by a size measurement, and this not does alter the relative positioning of the chromosomes.

**Figure 2:**
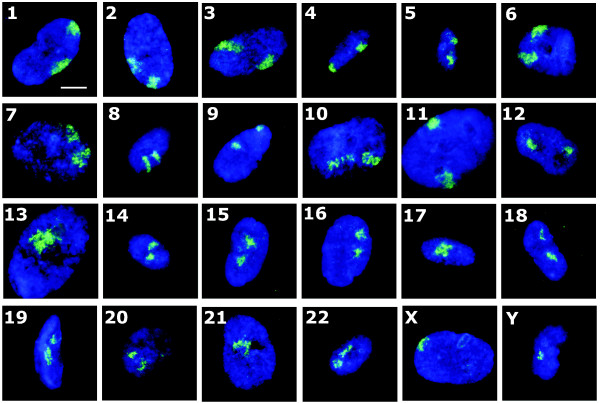
**Chromosome positioning in quiescent interphase nuclei**. Representative images displaying nuclei prepared for fluorescence *in situ *hybridization (2D-FISH), with whole-chromosome painting probes (green), and nuclear DNA is counterstained with 4',6-diamidino-2-phenylindole **(**DAPI) (blue). The cells were subjected to indirect immunofluorescence with anti-pKi-67 antibodies, and negative cells were selected. Cells were placed in low serum (0.5%) for 7 days, before fixation with methanol:acetic acid (3:1). The numbers (or letters) on the left side of each panel indicate the chromosome that has been hybridized. Scale bar = 10 μm.

**Figure 3:**
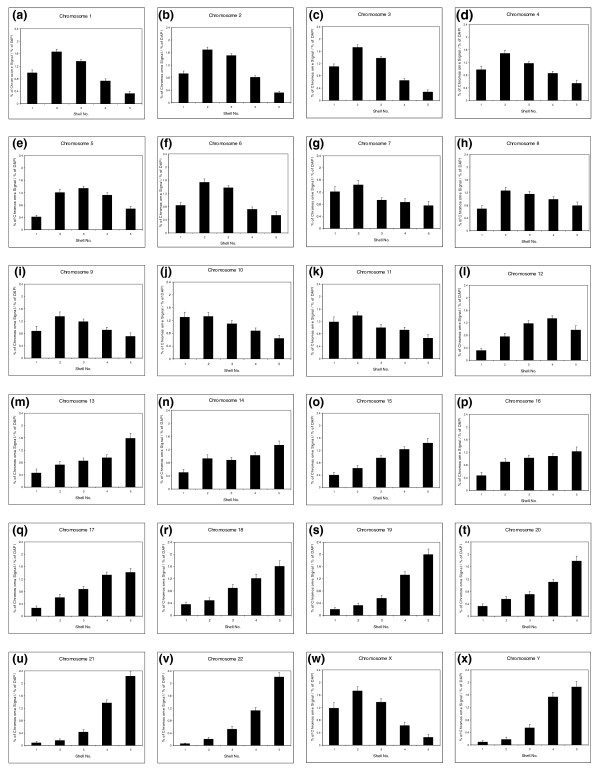
**Analysis of radial chromosome positioning in quiescent cell nuclei**. Histograms displaying chromosome positions in primary human quiescent fibroblast nuclei. The 50 to 70 nuclei per chromosome were subjected to erosion analysis, which divides each nucleus into five concentric shells of equal area, with shell 1 being the most peripheral shell, and shell 5 being the most interior shell [4-6,9]. The percentage of chromosome signal measured in each shell was divided by the percentage of 4',6-diamidino-2-phenylindole (DAPI) signal in that shell. Bars represent the mean normalized proportion (percentage) of chromosome signal for each human chromosome. Error bars represent the standard error of mean (SEM).

We have evidence from prior studies that chromosomes such as chromosomes 13 [9] and 18 [5,9] alter their nuclear position when primary fibroblasts exit the proliferative cell cycle and that chromosome X remains at the nuclear periphery [9]. However, this is only two chromosomes of 24, and so to determine which other chromosomes reposition after cell-cycle exit into quiescence (G_0_), elicited through serum removal, we positioned all human chromosomes in G_0 _cells (Figures 2 and 3).

To make cells quiescent, young, HDFs were grown in 10% NCS for 48 hours, and then the cells were washed twice with serum-free medium and placed in 0.5% NCS medium for 168 hours (7 days). However, when the positioning analysis was performed on the quiescent nuclei, we found that certain chromosomes were in very different positions from those in which they were found in proliferating nuclei, that is, chromosomes 1, 6, 8, 10, 11, 12, 13, 15, 18, and 20 (Table 1).

**Table 1:** The position of all chromosome territories in primary human dermal fibroblasts as determined by 2D FISH, image acquisition, and erosion analysis

Chromosome by size	Proliferating HDFs	Quiescent HDFs
**1**	IM^b^	P
**2**	P^b^	P
**3**	P^d^	P
**4**	P^cd^	P^c^
**5**	IM^d^	IM
**6**	IM^b^	P
**7**	P^	P
**X**	P^ab^	P^c^
**8**	IM^b^	P
**9**	P^d^	P
**10**	IM^d^	P
**11**	IM^d^	P
**12**	P^b^	I
**13**	P^a^	I^c^
**14**	I^b^	I
**15**	P^b^	I
**16**	I^b^	I
**17**	I^b^	I
**18**	P^ac^	I^ac^
**19**	I^a^	I^a^
**20**	I^d^	IM
**22**	I^b^	I
**21**	I^b^	I
**Y**	I^	I

This table summarizes the locations of all the chromosomes in quiescent and proliferating nuclei of human dermal fibroblasts (HDFs). The positions of chromosomes shown without a symbol have been determined for this study. ^a^Data derived from [5]. ^b^Data derived from [4]. ^c^Data derived from [9]. ^d^Data derived from [7].

The data demonstrated in Figure 3 and Table 1 reveal that a number of chromosomes alter their nuclear positions when cells become quiescent; as shown before, both chromosomes 13 and 18 move from a peripheral nuclear location to an interior location (Figure 3m and r). Chromosome 10 is one of a number of chromosomes that move from an intermediate nuclear location to the nuclear periphery (Figure 3j, Table 1), whereas chromosome X does not change its location at the nuclear periphery (Figure 3w), and chromosomes such as 17 and 19 do not change their interior location (Figure 3q and s, respectively).

It certainly appears that the chromosome positioning in quiescent G_0 _cells is correlated with size. However, it is not clear why a repositioning of chromosomes occurs after serum removal and when and how it is elicited.

### The movement of chromosomes when normal fibroblasts exit the cell cycle is rapid, active, and requires myosin and actin

**Figure 4:**
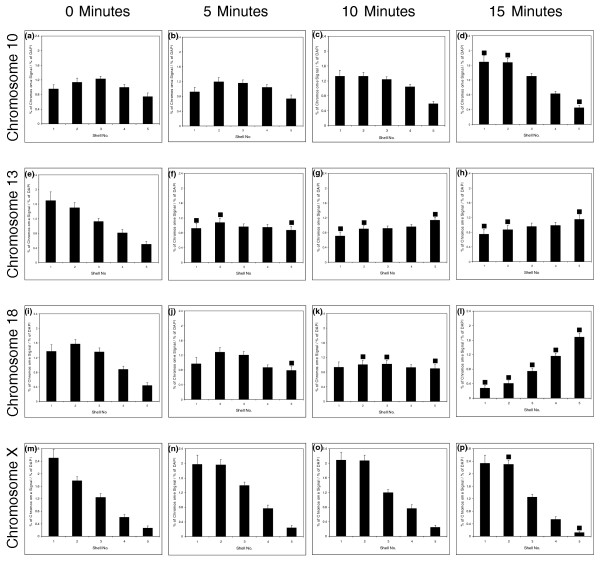
**Rapid repositioning of chromosomes after removal of serum**. Chromosomes move rapidly in proliferating cells placed in low serum. The nuclear locations of human chromosomes 10 **(a-d)**, 13 **(e-h)**, 18 **(i-l)**, and X **(m-p) **were analyzed in normal fibroblast cell nuclei fixed for 2D-FISH (fluorescence *in situ *hybridization) after incubation in medium containing low serum (0.5%) for 0, 5, 10, and 15 minutes. The filled-in squares indicate significance difference (*P *< 0.05) when compared with control proliferating fibroblast cell nuclei.

To determine when the genome is reorganized on exit from the cell cycle and the speed of the response to the removal of growth factors, we took actively proliferating young cultures of primary HDFs and replaced 10% NCS medium with 0.5% NCS medium. Samples were taken at 0, 5, 10, 15, and 30 minutes after serum starvation for fixation, and chromosome position in interphase was determined with 2D-FISH and erosion analysis (Figure 4 and Additional file 1). Chromosomes 13 and 18 relocated from the nuclear periphery to the nuclear interior within 15 minutes (Figure 4h and l), with an intermediate-type nuclear positioning visible in the intervening time points (5 and 10 minutes; Figure 4f, g, j, and k). In addition, chromosome 10 moved from an intermediate location to a peripheral location in the same time window (15 minutes; Figure 4d). Chromosome X did not relocalize at all, as was reported previously [9] (Figure 4m-o), apart from some slight difference at 15 minutes (Figure 4p).

**Figure 5:**
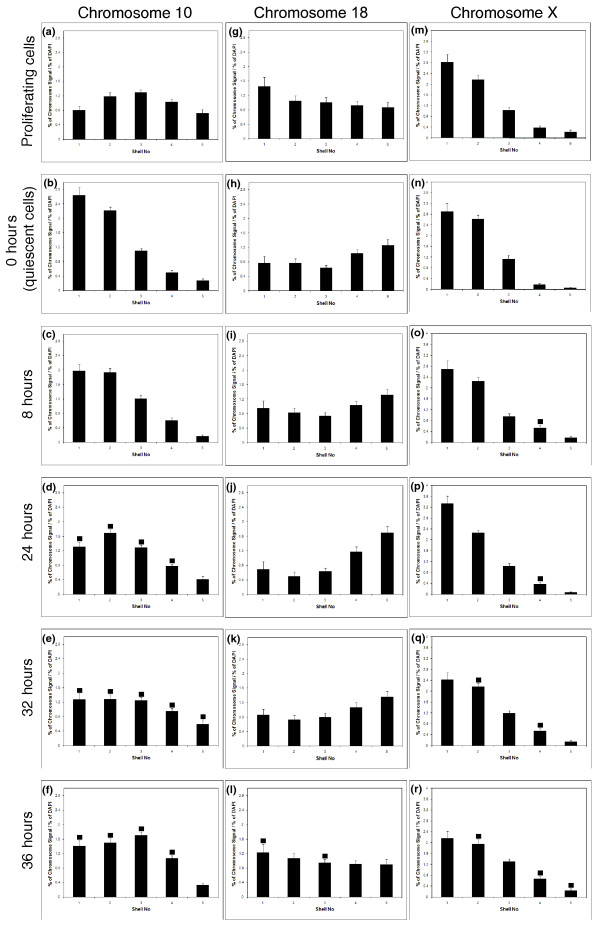
**Restoration of proliferative chromosome position after restimulation of G_0 _cells**. The relocation of chromosomes to their proliferative nuclear location takes 24+ hours for chromosome 10 and 36 hours for chromosome 18. Proliferating cells (**a, g, m**) were placed in low serum (0.5%) for 7 days (**b, h, n**) and then restimulated to enter the proliferative cell cycle with the readdition of high serum. Samples were taken at 8 hours (**c, i, o**), 24 hours (**d, j, p**), 32 hours (**e, k, q**), and 36 hours (**f, l, r**) after restimulation. The graphs display the normalized distribution of chromosome signal in each of the five shells. Shell 1 is the nuclear periphery, and shell 5 is the innermost region of the nucleus. The solid squares represent a significant difference (*P *< 0.05) for that shell when compared with the equivalent shell for the time 0 data (G_0 _data) for the erosion analysis.

In a previous study, we demonstrated that relocation of chromosome 18 from the nuclear interior in G_0 _cells to the nuclear periphery in serum-restimulated cells took 30+ hours and appeared to require cells to rebuild their nuclear architecture after a mitotic division [5]. We showed here that the same is true for chromosome 10, with a return to an intermediate nuclear location 24 to 36 hours after restimulation of G_0 _cells with 10% NCS (Figure 5d-f). We again showed that chromosome 18 requires similar times to return to the nuclear periphery (that is, 36 hours; Figure 5l). Although chromosome X remains at the nuclear periphery, a slight change in the distribution of chromosome X occurs at 32 to 36 hours (Figure 5q-r). From these data, it seems that a rapid response to the removal of growth factors reorganizes the whole genome within the interphase nucleus, and this reorganization is corrected in proliferating cells only after 24+ hours in high serum, presumably after the cells have passed through mitosis, as indicated by the peak of mitotic cells at 24 to 36 hours after serum restimulation (0 hours, none; 8 hours, none; 24 hours, 0.3%; 32 hours, 2.6%; and 36 hours, 1.2%).

**Figure 6:**
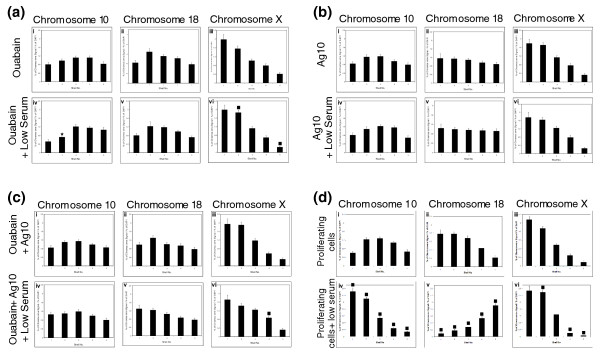
**Chromosome repositioning requires energy**. The relocation of human chromosomes 10 and 18 after incubation in low serum is energy dependent. The nuclear location of human chromosomes 10, 18, and X in were determined in normal human proliferating cell nuclei treated with ouabain (ATPase inhibitor) **(a)**, AG10 (GTPase inhibitor) **(b)**, or a combination of both **(c) **before and during incubation in low serum for 15 minutes. Normal control analysis data without any treatment is displayed in **(d)**. The error bars show the standard error of the mean. The stars indicate a significant difference (*P *< 0.05) from cells treated only with the inhibitor.

**Figure 7:**
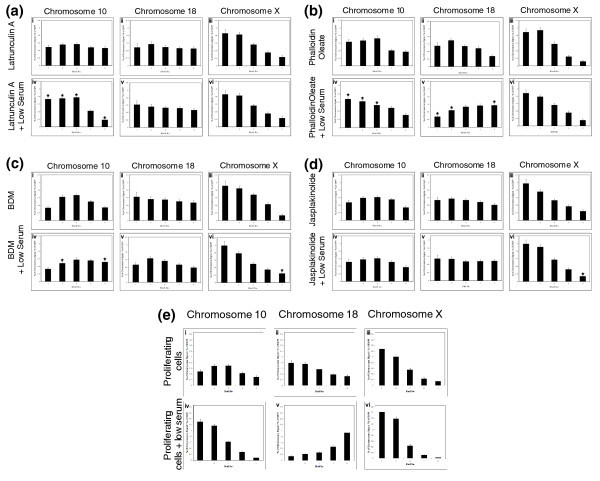
**Chromosome repositioning requires nuclear myosin and actin**. The relocation of human chromosomes 10 and 18 after incubation in low serum is myosin and actin dependent. The nuclear locations of chromosomes 10, 18, and X were determined in normal human proliferating cell nuclei treated with latrunculin A and phalloidin oleate (inhibitors of actin polymerisation) **(a, b) **and BDM and jasplakinolide (inhibitors of myosin polymerization) **(c, d) **before and during incubation in low serum for 15 minutes. The error bars show the standard error of the mean. The stars indicate a significant difference (*P *< 0.05) from cells treated only with the inhibitor. Normal control analysis data without any treatment is displayed in **(e)**.

Such rapid movement of large regions of the genome in response to low serum implies an active process, perhaps requiring ATP/GTP. When inhibitors of ATPase and/or GTPase, ouabain, and AG10, were incubated with proliferating cell cultures in combination with low serum, chromosome 10 did not change nuclear location (Figure 6a-d, and see Additional file 3). The relocation to the nuclear interior of chromosome 18 territories after incubation of cells in low serum also was perturbed by these inhibitors (Figure 6a-d). The control chromosome, chromosome X, remained at the nuclear periphery (Figure 6 and Additional file 3). Because other studies suggest that nuclear motors move genomic regions around the nucleus by actin and/or myosin [42,44] we decided to use inhibitors of actin and myosin polymerization to attempt to block any chromosome movement elicited by these nuclear motors when serum was removed. Latrunculin A, an inhibitor of actin polymerization, inhibited the movement of both chromosomes 10 and 18 when cells were placed in low serum (Figure 7a and Additional file 3). In contrast, phalloidin oleate, another inhibitor of actin polymerization did not prevent relocalization of either chromosome 10 or 18, when cells were placed in low serum (Figure 7b and Additional file 3). However, two inhibitors of myosin polymerization (BDM) and function (Jasplakinolide; also affects actin polymerization) did inhibit movements of both these chromosomes upon serum removal (Figure 7c, d, and Additional file 3). Figure 7e provides a comparison for the rapid change in chromosome position when no inhibitors are used. These data imply that rapid chromosome movement observed in cells as they respond to removal of growth factors is due to an energy-driven process involving a nuclear actin:myosin motor function.

### Nuclear myosin 1β is required for chromosome territory repositioning in HDFs placed in low serum

**Figure 8:**
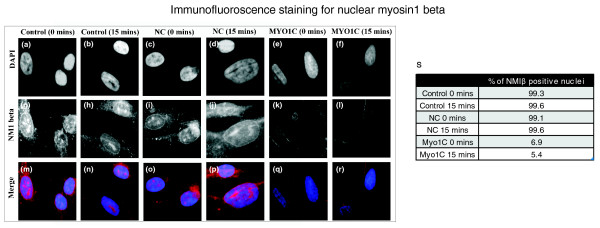
**Suppression of nuclear myosin expression by short interference RNAs (siRNAs)**. Normal human dermal fibroblasts (HDFs) were transfected with negative control or MYO1C targeting siRNA (double transfection) and samples for immunofluorescence staining and 2D-FISH (fluorescence *in situ *hybridi**z**ation) were fixed 48 hours after the final transfection. Representative images of nuclei stained for anti-NMIβ (red) in control **(g, h, m, n) **cells transfected with negative control siRNA **(i, j, o, p) **and in cells transfected with *MYO1C *siRNA **(k, l, q, r) **after 0 and 15 minutes of serum starvation are displayed. The percentage of nuclei that are positive for NM1β in controls, in cells transfected with negative control siRNA, and in cells transfected with *MYO1C *siRNA are displayed in the adjacent table **(s)**.

In an effort to elucidate which myosin isoform was involved in chromosome movement after serum removal in culture, we used suppression by RNA interference with small interfering RNAs (siRNAs). An siRNA pool for the gene *MYO1C *was selected because it encodes for a cytoplasmic myosin 1C and the nuclear isoform nuclear myosin 1β, a major candidate myosin for chromatin relocation [39,49]. mRNA analysis had revealed insufficient differences in sequence for suppression of myosin 1β alone (data not shown). With a double transfection of the siRNA, we observed 93% of cells displaying no nuclear myosin staining at all (Figure 8k, q, and s) but still with some cytoplasmic staining, whereas in the control cells and the cells transfected with the control construct, >95% of cells displayed a nuclear distribution of anti-nuclear myosin 1β, which was distributed in proliferating cells as accumulations at the inner nuclear envelope, the nucleoli, and throughout the cytoplasm (Figure 8g-j, m-p). These numbers did not change significantly after serum removal for 15 minutes, as per the chromosome-movement assay (data not shown).

**Figure 9:**
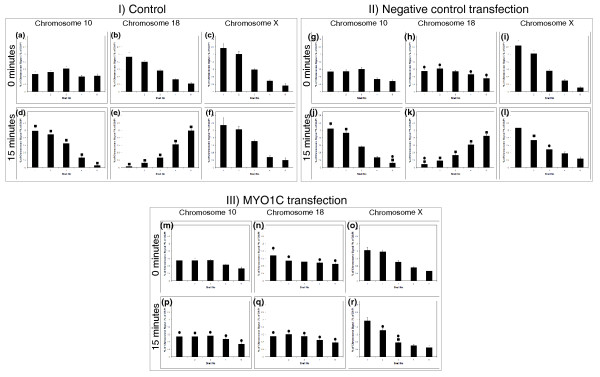
**Chromosome repositioning is inhibited by short interference RNA (siRNA) that suppresses nuclear myosin1β**. Chromosome positioning was determined with 2D-FISH (fluorescence *in situ *hybridi**z**ation) and erosion analysis, and the normalized position data plotted as histograms in control cells, in cells transfected with the negative control, and in cells transfected with the *MYO1C *siRNA construct. In control human dermal fibroblasts (HDFs) and in HDFs transfected with negative control, siRNA chromosome 10 is repositioned from an intermediate nuclear location **(a **and **g, respectively) **to the nuclear periphery **(d, j) **after 15 minutes of incubation in low serum. Chromosome 18 territories, conversely, are repositioned from the nuclear periphery **(b, h) **to the nuclear interior **(e, k) **after 15 minutes of incubation in low serum in control HDFs and in HDFs transfected with negative control siRNA. In HDFs transfected with the *MYO1C *siRNA construct, chromosomes 10 **(m, p) **and 18 **(n, q) **do not show repositioning after 15 minutes of incubation in low serum. Chromosome X remains at the nuclear periphery at all times **(c, f, i, l, o, r)**. Unpaired, unequal variances two-tailed Students *t *tests were performed to assess statistical differences. The solid squares indicate a significant difference (*P *< 0.05) from cells not incubated in, and the solid circles indicate a significant difference (*P *< 0.05) from control HDFs.

After siRNA suppression of nuclear myosin, the chromosome-movement assay was repeated by placing the double-transfected cells into low serum for 15 minutes. The graphs in Figure 9 show that chromosomes 10, 18, and X behave as expected after removal of serum in the control cells (Figure 9a-f) and in the cells transfected with the control construct (Figure 9g-l), with chromosome 10 becoming more peripheral, chromosome 18 becoming more interior, and chromosome X remaining at the nuclear periphery. However, in the cells that had been transfected with *MYO1C*-targeting siRNA, chromosome movement was much less dramatic, with the chromosomes still residing in similar nuclear compartments before and after the serum removal (Figure 9m-r).

**Figure 10:**
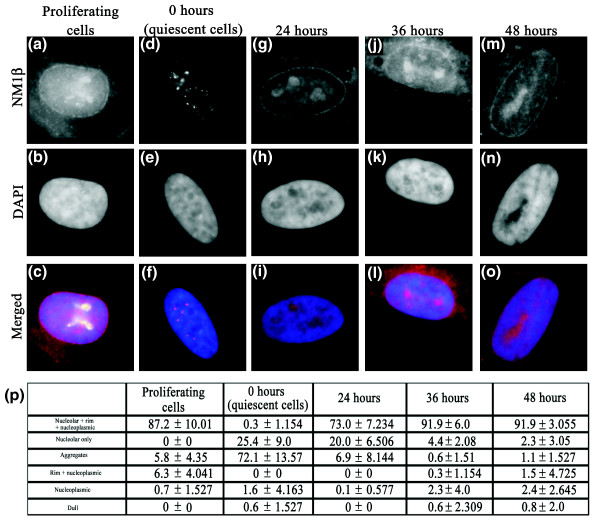
**Anti-nuclear myosin 1b (NM1β) staining patterns in proliferating cells, quiescent cells, and after restimulation**. Normal 2DD human dermal fibroblasts (HDFs) were serum starved for 7 days to induce quiescence. The cells were then restimulated with fresh serum, and samples were collected at 0, 24, 36 and 48 hours after serum restoration. Samples were also collected before serum withdrawal (proliferating cells). The samples were then fixed with methanol/acetone (1:1), and the distribution of NMIβ was assessed by performing an indirect immunofluorescence assay for NMIβ. Images in **(a, c) **display the distribution of NMIβ in proliferating cells, whereas those in **(d **and **f) **show the distribution of NMIβ after 0, 24, 36 and 48 hours after restimulation of quiescent fibroblasts. The table **(p) **displays the percentage of cells displaying various patterns of NMIβ staining after restimulation. Error is indicated by standard deviation. Scale bar = 10 μm.

The distribution of the nuclear myosin 1β is very interesting in these cells, because it gives a nuclear envelope distribution, a nucleolar distribution, and a nucleoplasmic distribution (Figure 10a-c). These distributions, although revealing, are not so surprising, because nuclear myosin has a binding affinity for the integral nuclear membrane protein emerin [50] and is involved in RNA polymerase I transcription [37,40,51]. The distribution in quiescent cells is quite different, with large aggregates of NM1β within the nucleoplasm and is missing from the nuclear envelope and nucleoli. This distribution is similar to that observed in senescent human dermal fibroblasts (Mehta, Kill, and Bridger, unpublished data). With respect to chromosome movement back to a proliferating position after incubation in low serum, we showed that it does not occur until 24 to 36 hours after repeated addition of serum to a quiescent culture (Figure 5) [5]. Correlating with this is the rebuilding of daughter nuclei after mitosis and the return of a proliferating distribution of NM1β to the nuclear envelope, nucleoli, and nucleoplasm (Figure 10g, j, p).

## Discussion

This study completes the nuclear positioning of all 24 chromosomes in quiescent (serum-starved) normal primary HDFs, as assessed with 2D-FISH and erosion analysis, with a number of chromosomal positions confirmed in 3D-preserved nuclei. This study, which was performed on similar cell cultures and in the same way as previous studies, highlighted that some considerable difference exists in chromosomal nuclear locations between proliferating and quiescent cells. This difference cannot be due to change in nuclear size or shape, because some chromosomes move toward the nuclear interior, some, to the nuclear periphery, and some do not alter their location at all; no significant difference is found between nuclear shape and size before and after 15 minutes in low serum (data not shown). Some suggestion exists of a size-correlated distribution in quiescent cells (Table 1), with large chromosomes toward the nuclear periphery, and small chromosomes toward the nuclear interior. These results also confirm the data previously presented, whereby small chromosomes 13 and 18 had differential nuclear locations with respect to proliferating and nonproliferating cells [5,9].

How and when the alterations to chromosome positioning occur are two fundamental questions in understanding the role of genome organization in cell cycle-related events. The genome is probably anchored and influenced through a number of interactions with nuclear architecture [52,53], and so any signalled alterations/modifications to these structures could enable a reorganization of the position of chromosome territories. We know that when cells are made quiescent (for 7 days) and are restimulated to enter the cell cycle by the repeated addition of serum, chromosome 18 is not relocated back to the nuclear periphery until the cells have been through mitosis [5].

The question remained open as to when chromosomes were repositioned after serum removal. We found that repositioning of chromosomes was very rapid and complete by 15 minutes. The types of repositioning (a) requiring a rebuilding of the nucleus after mitosis, and (b) the rapid response without a nuclear envelope breakdown, imply that these processes follow different pathways and mechanisms, and the latter is consistent with an energy-dependent mechanism. This rapid movement of chromosomes after growth factor removal may be elicited through a nuclear motor such as the actin/myosin motor-complex, containing nuclear actin and nuclear myosin I, previously shown to be involved in intranuclear movements of chromatin [42-44]. This hypothesis was supported by experiments using inhibitors of ATPase and GTPase, as well as inhibitors of actin and myosin polymerization. The actin polymerization inhibitor phalloidin oleate did not inhibit chromosome movement on removal of high serum. This is important because phalloidin has been shown not to bind to nuclear actin unless the cells are treated with DMSO [54], which we had not done.

These data support other literature describing nuclear motors being involved in chromatin behavior [44]. These drugs have an effect on a broad range of myosins, and so we wanted to assess whether specific myosins were involved; thus we used an siRNA sequence that successfully suppressed the levels of nuclear myosin 1β, as shown by indirect immunofluorescence. This is the only nuclear myosin that would have been affected, but we cannot rule out that other myosins located within the cytoplasm (such as myosin 1A and 1C), which may be suppressed as well, could have a long-range interaction with chromatin, through the nuclear envelope, possibly through nesprins and SUN domain proteins [55,56].

However, the distribution of nuclear myosin 1β that we observe in proliferating cells correlates with its properties and functions, as described in the literature, and implicates the nuclear envelope in chromosome/chromatin movement. In previous studies, we analyzed chromosome position in cells that have defects of the nuclear lamina, through mutations in nuclear lamin A or emerin, both nuclear envelope proteins. These cells displayed a nonproliferating distribution of chromosomes even though they were proliferating [9,57]. The behavior of nuclear motor proteins in these cells must be addressed. Further, the distribution of NM1β from aggregates in quiescent cells to the nuclear envelope, nucleoli, and nucleoplasm is not observed until more than 24 hours after serum readdition, which correlated with when specific chromosomes become relocated from their quiescent position to their proliferating location.

## Conclusions

We demonstrated that some chromosomes occupy different nuclear compartments in proliferating and serum-starved quiescent cells. Most interestingly, this repositioning of chromosomes is very rapid, taking less than 15 minutes, and requires energy and active actin and myosin function. The myosin involved could be nuclear myosin 1β, which has dramatically different distribution in quiescent nuclei as compared with proliferating cell nuclei.

## Materials and methods

### Cell culture

Human dermal fibroblasts (HDFs), 2DD [58] were grown in Dulbecco's Modified Eagles Medium (DMEM) containing 10% newborn calf serum (vol/vol NCS), glutamine, and antibiotics, at 37°C. Cells were passaged twice a week and seeded at a density of 3 × 10^3^/cm^2^. Cells were made quiescent by incubation in 10% NCS DMEM for 2 days, washing in serum-free medium, followed by incubation in DMEM containing 0.5% NCS (vol/vol) for 7 days.

### Inhibitors of ATPase, GTPase, myosin, and actin polymerization

To inhibit the activity of ATPase or GTPase, cells were treated with 100 μmol/L ouabain (Calbiochem-Novabiochem, Beeston, Nottingham, UK) for 30 minutes [59] or with 100 μmol/L AG10 (Calbiochem) for 20 or 30 minutes before serum withdrawal [60,61], respectively. To inhibit the polymerization of actin, cells were treated with 1 μmol/L either Latrunculin A (Calbiochem) [62,63] or phalloidin oleate (Calbiochem) [64] for 30 minutes. Myosin polymerization was inhibited by treating cells either with 10 mmol/L 2,3-butanedione 2-monoxime (Calbiochem) for 15 minutes [65-67] or 1 μmol/L Jasplakinolide (Calbiochem) for 60 minutes [68]. See Additional file 5.

### Fluorescence in situ hybridization

For the two-dimensional FISH assay, fibroblasts were harvested and placed in hypotonic buffer (0.075 mol/L KCl, wt/vol) for 15 minutes at room temperature. After centrifugation at 400 g, cells were fixed in 3:1 (vol/vol) methanol/acetic acid (vol/vol) for 1 hour on ice. The fixation step was repeated between 5 and 7 times before cells were dropped onto humidified glass microscope slides. The slides were aged at room temperature for 2 days or for an hour at 70°C before being subjected to dehydration through an ethanol series of 70%, 90%, and 100%, for 5 minutes each. The cells were denatured in 70% formamide, 2 × sodium saline citrate buffer (SSC), pH 7, at 70°C for 2 minutes. After denaturation, the slides were immediately plunged into ice-cold 70% ethanol for 5 minutes and then taken through the ethanol series and air-dried.

For three-dimensional FISH assay, fibroblasts were washed in 1 × PBS and then fixed in 4% paraformaldehyde (wt/vol) in 1 × PBS for 10 minutes. The cells were then permeabilized with 0.5% Triton-X100 (vol/vol) and 0.5% saponin (wt/vol) in 1 × PBS solution for 20 minutes. The cells were then incubated in 20% glycerol, 1 × PBS solution for at least 30 minutes before being snap-frozen in liquid nitrogen. The cells were repeatedly frozen and thawed for up to 5 times. After the freeze/thaw cycles, the cells were then washed in 1 × PBS for at least 30 minutes and then incubated in 0.1 N HCl for 10 minutes for depurination. The cells were then washed in 2 × SSC for 15 minutes, with three changes of the buffer, and incubated in 50% formamide, 2 × SSC, at pH 7.0, overnight. For denaturation, cells were incubated at 73°C to 76°C in 70% formamide, 2 × SSC, pH 7 solution for 3 minutes and then were immediately transferred to 50% formamide, 2 × SSC, pH 7 solution for 1 minute at the same temperature. Chromosome paints for HSA 10, 13, 18, and X were amplified from flow-sorted whole-chromosome templates and labelled with biotin-dUTP by DOP-PCR [69]. The 200- to 400-μg chromosome paints, 7 μg of C_0_*t-*1 DNA, and 3 μg of herring sperm were used per slide. All other chromosome territories were delineated with directly labelled whole human chromosome paints (Qbiogene, Cambridge, UK). Probes were denatured at 70°C for 10 minutes with reannealing of repetitive sequences at 37°C for 30 to 120 minutes. Hybridization was performed in a humified chamber for 18 to 24 hours at 37°C. The slides were washed in three changes of 50% formamide, 2 × SSC, pH 7, at 45°C over a 15-minute period, followed by three changes of 0.1 × SSC prewarmed to 60°C over a 15-minute period at 45°C.

The slides were then transferred to 4 × SSC at room temperature. Slides hybridized with the in-house biotin-labelled probes were then incubated with a blocking solution of 4% bovine serum albumin (BSA; Sigma Aldrich) of 4 × SSC followed by detection with streptavidin-cyanine 3 (Amersham Life Science Ltd; 1:200 dilution in 0.1% BSA/4 × SSC). The slides were washed in three changes of 4 × SSC/0.05% Tween 20 (vol/vol) for 5 minutes each.

### Suppressing the expression of nuclear myosin 1β by interference RNA

To suppress nuclear myosin 1β expression, young proliferating HDFs were seeded at 1 × 10^4 ^cells per well in a 12-well plate. Transfection efficiency was previously determined with siGLO-labelled siRNA to be more than 95%. The siRNA transfection was carried out with 2 μl Dharmafect 1 and 50 μl of either negative control (2 μmol/L ON-TARGETplus Non-targeting Pool; Thermo Scientific) or myosin-targeting siRNA (2 μmol/L ON-TARGETplus SMART pool, human MYO1C; Thermo Scientific Cat number L-015121-00) in 200 μl serum-free medium. Complete medium was added to the transfection mix to ensure that transfections were carried out in serum-containing medium with a final siRNA concentration of 100 nmol/L per well/dish. Six hours after transfection, the medium in the well was replaced with normal growth medium. At 24 hours after the first transfection, a second identical transfection was performed to increase the amount of suppression. Samples were collected at 48 hours after final transfection and fixed for 2D FISH and indirect immunofluorescence.

### Indirect immunofluorescence

Diluted rabbit anti-Ki-67 antibody (Dako; 1:1,500 dilution in PBS/1% NCS), 40 μl, was placed on the slides after FISH for 1 hour at 37°C. Slides were washed in PBS for 15 minutes, with three changes. The slides were incubated with 40 μl of swine anti-rabbit secondary antibody conjugated either to fluorescein isothiocynate (FITC, Dako) or to tetrarhodamine isothiocynate (TRITC, Dako) (1:30 dilution in 1% NCS/PBS) for 1 hour at 37°C.

For anti-nuclear myosin 1β staining, cells were grown on glass coverslips and fixed in 1:1 (vol/vol) methanol/acetone for 10 minutes on ice. Rabbit anti-NM1β (Sigma-Aldrich) was diluted in PBS/1% NCS (1:200) and incubated with the fixed cells at room temperature for 1 hour after washing thrice in PBS swine anti-rabbit conjugated to tetrarhodamine isothiocyanate was incubated for 1 hour at room temperature.

Thereafter the slides were washed in PBS with three changes over a 15-minute period and mounted in self-sealing Vectashield mounting medium (Vector Laboratories) containing the counterstain 4, 6-diamidino-2-phenylindole (DAPI).

### Image capture and analysis

#### Two-dimensional FISH

Digital grey-scale images of random nuclei were captured by using a Photometrics cooled charged-coupled device (CCD) camera, pseudocolored, and merged by using Digital Scientific software, the Quips Pathvysion, Smart Capture VP V1.4, a Leica fluorescence microscope (Leitz DMRB) with Plan Fluotar 100 × oil-immersion lens. The images were run through a simple erosion script in IPLab spectrum software, as described in [4]. The DAPI image of the nucleus is outlined and divided into five concentric shells of equal area, the first shell being most peripheral, and the innermost denoting the interior of the nucleus. The script measures the pixel intensity of DAPI and the chromosome probe in these five shells. The probe signal was normalized by dividing the percentage of the probe by the percentage of DAPI signal in each shell. Histograms were plotted with standard error bars representing the standard error of the mean (± SEM). Simple statistical analyses were performed by using the unpaired two-tailed Student's *t *test with Microsoft Excel.

#### Three-dimensional FISH

The images of nuclei prepared by three-dimensional FISH were captured by using a Nikon confocal laser scanning microscope (TE2000-S) equipped with a 60 ×/1.49 Nikon Apo oil-immersion objective. The microscope was controlled by Nikon confocal microscope C1 (EZ-C1) software, version 3.00. Stacks of optical sections with an axial distance of 0.2 μm were collected from 20 random nuclei. Stacks of eight-bit grey-scale 2D images were obtained with a pixel dwell of 4.56, and eight averages were taken for each optical image. The positioning of chromosomes in relation to the nuclear periphery was assessed by performing measurements with Imaris Software (Bitplane Scientific Solutions), whereby the distance in micrometers between the geometric center of each chromosome territory and the nearest nuclear periphery, as determined with DAPI staining, in three dimensions. These data were not normalized for size, but when the data were normalized by dividing by the length of the major axis + the length of the minor axis divided by 2, or the length of the major axis alone, the relative positions of the individual chromosomes in frequency distributions did not change.

Frequency distribution curves were plotted with the distance between the geometric center of chromosome territory and the nearest nuclear periphery on the x-axis in actual micrometers, and the frequency, on the y-axis.

## Abbreviations

BDM: 2,3-butanedione 2-monoxime; FITC: fluorescein isothiocyanate; G_0_: quiescence; HDF: human dermal fibroblast; I: interior; IM: intermediate; NCS: newborn calf serum; NMIβ: nuclear myosin Iβ; P: peripheral; TRITC: tetrarhodamine isothiocyanate.

## Authors' contributions

ISM provided material, experimentation, data collection and analysis, writing manuscript, and intellectual input. MA provided some data for nuclear shape and size and some intellectual input for siRNA. AH provided intellectual input for siRNA experimentation and writing of the manuscript. JMB participated in data analysis, writing the manuscript, and intellectual input.

## Supplementary Material

Additional data file 1 The chromosome position of chromosomes 10, 13, 18, and X 30 minutes after serum removal from a proliferating culture of human dermal fibroblasts in a 2D study (1A-D), and the 3D analysis of the nuclear position of chromosomes 10 and X after 15 minutes after serum removal from a proliferating culture (1E). Click here for file

Additional data file 2 Treating cells with 0.1% DMSO, in which the drugs are dissolved, does not interfere with the chromosome-repositioning response. Click here for file

Additional data file 3 3D analyses of chromosome position for chromosomes 10 and X after treatment with GTPase inhibitor AG10 and serum removal (3A), after treatment with phalloidin oleate and serum removal (3B) and after treatment with BDM and serum removal (3C). Click here for file

Additional data file 4 The DAPI distribution with each shell of the 2D erosion analysis script for each experiment performed, revealing that the DNA content did not alter after any of the treatments (4). Click here for file

Additional data file 5 A table describing the inhibitors and drugs used in this study. Click here for file
