# Supplementary material for: Rapid chromosome territory relocation by nuclear motor activity in response to serum removal in primary human fibroblasts
Source: Genome Biol. 2010 Jan 13;11(1):R5. doi: 10.1186/gb-2010-11-1-r5 (PMC2847717; doi:10.1186/gb-2010-11-1-r5)
Supplement: Additional data file 1 — The chromosome position of chromosomes 10, 13, 18, and X 30 minutes after serum removal from a proliferating culture of human dermal fibroblasts in a 2D study (1A-D), and the 3D analysis of the nuclear position of chromosomes 10 and X after 15 minutes after serum removal from a proliferating culture (1E). [file gb-2010-11-1-r5-S1.pdf]

Figure S1

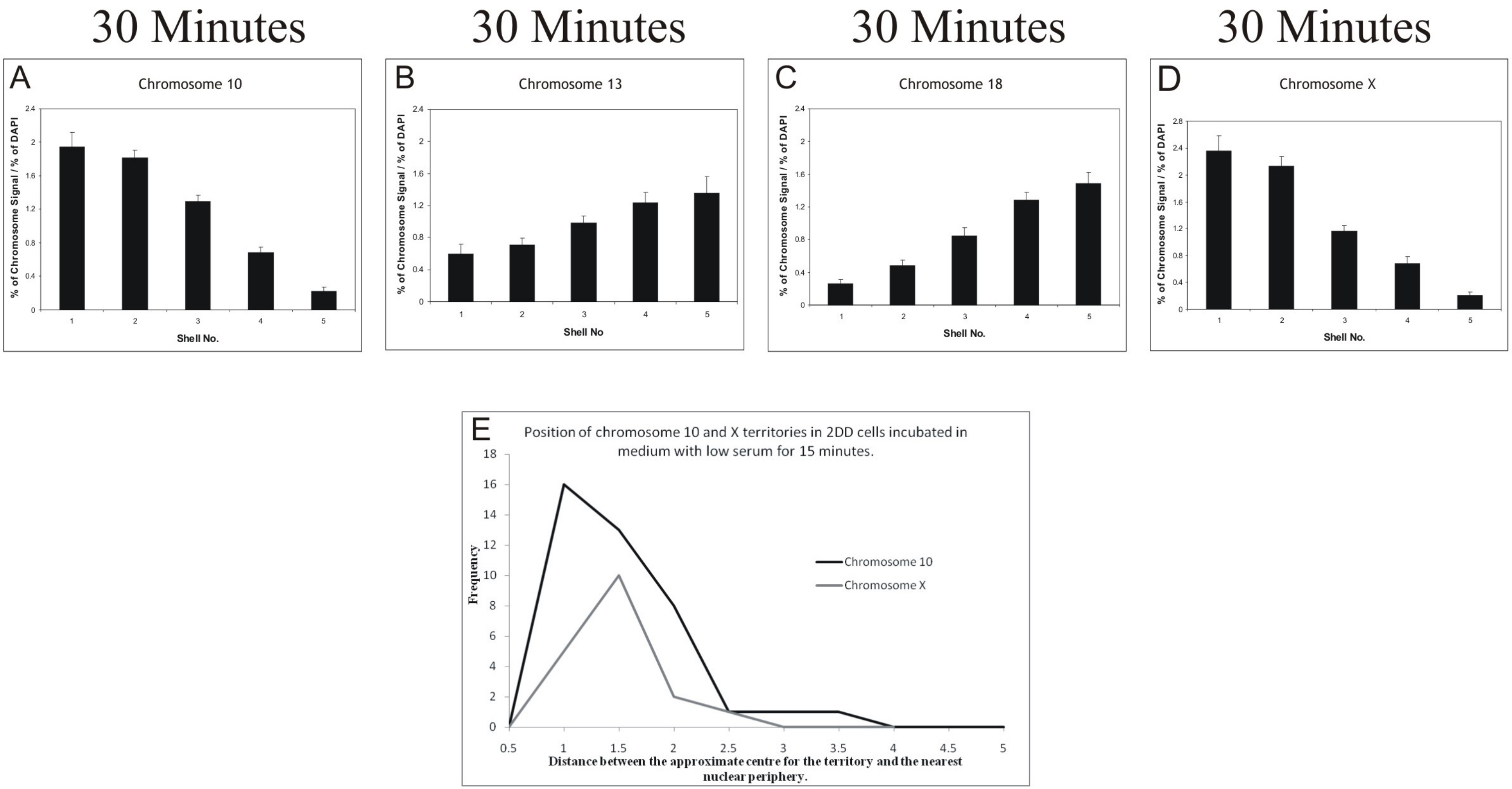

Figure 1 : The nuclear locations of human chromosome 10, 13, 18 and X territories were analysed in normal fibroblast cell nuclei after incubation of cells in medium containing low serum for 30 minutes. The percentage of chromosome signal measured in each shell was divided by the percentage of DAPI signal in that shell. Bars represent the mean normalised proportion (%) of chromosome signal for each human chromosome (A, B, C and D). Error bars represent standard error of mean (SEM) Panel E displays the location of chromosome 10 and X territories respectively in fibroblasts incubated in medium with low serum for 15 minutes in form of a frequency distribution curve obtained by plotting measurements from 3D-FISH data. Unpaired, unequal variance, two-tailed student's t-test at 95% confidence interval ($p < 0.05$) has shown that there is a significant difference between the position of chromosome 10 in normal proliferating fibroblasts and fibroblasts incubated in low serum for 15 minutes. While the position of chromosome X in cells subjected to both of these conditions do not show any significant difference.
