# Supplementary material for: Rapid chromosome territory relocation by nuclear motor activity in response to serum removal in primary human fibroblasts
Source: Genome Biol. 2010 Jan 13;11(1):R5. doi: 10.1186/gb-2010-11-1-r5 (PMC2847717; doi:10.1186/gb-2010-11-1-r5)
Supplement: Additional data file 2 — Treating cells with 0.1% DMSO, in which the drugs are dissolved, does not interfere with the chromosome-repositioning response. [file gb-2010-11-1-r5-S2.pdf]

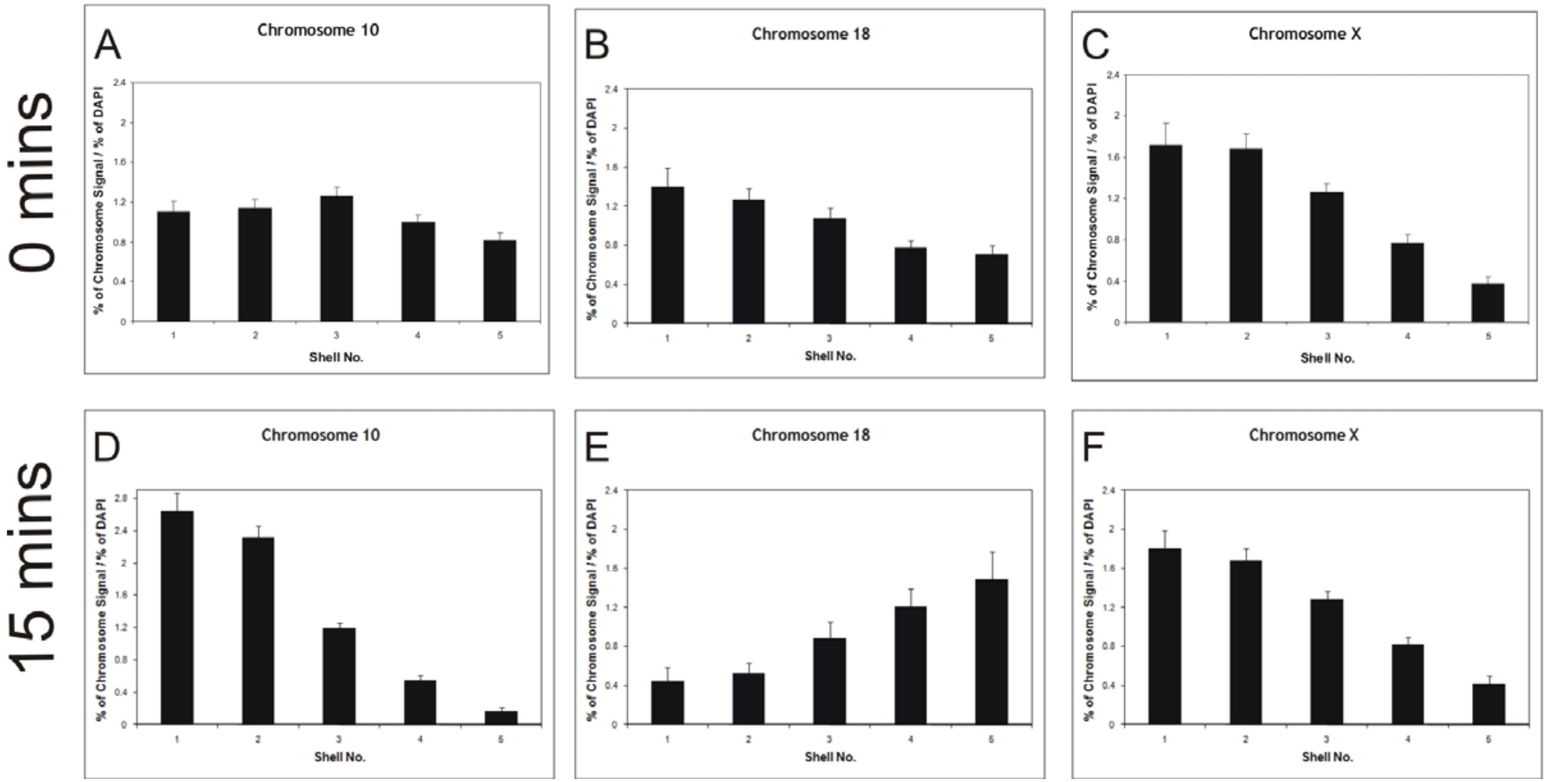

Figure 2: The nuclear locations of human chromosome 10, 18 and X territories were determined in normal human proliferating cell nuclei treated with 0.1% DMSO before and during incubation in low serum for 15 minutes. The error bars show standard error of mean.
