# Supplementary material for: Rapid chromosome territory relocation by nuclear motor activity in response to serum removal in primary human fibroblasts
Source: Genome Biol. 2010 Jan 13;11(1):R5. doi: 10.1186/gb-2010-11-1-r5 (PMC2847717; doi:10.1186/gb-2010-11-1-r5)
Supplement: Additional data file 3 — 3D analyses of chromosome position for chromosomes 10 and X after treatment with GTPase inhibitor AG10 and serum removal (3A), after treatment with phalloidin oleate and serum removal (3B) and after treatment with BDM and serum removal (3C). [file gb-2010-11-1-r5-S3.pdf]

Figure S3

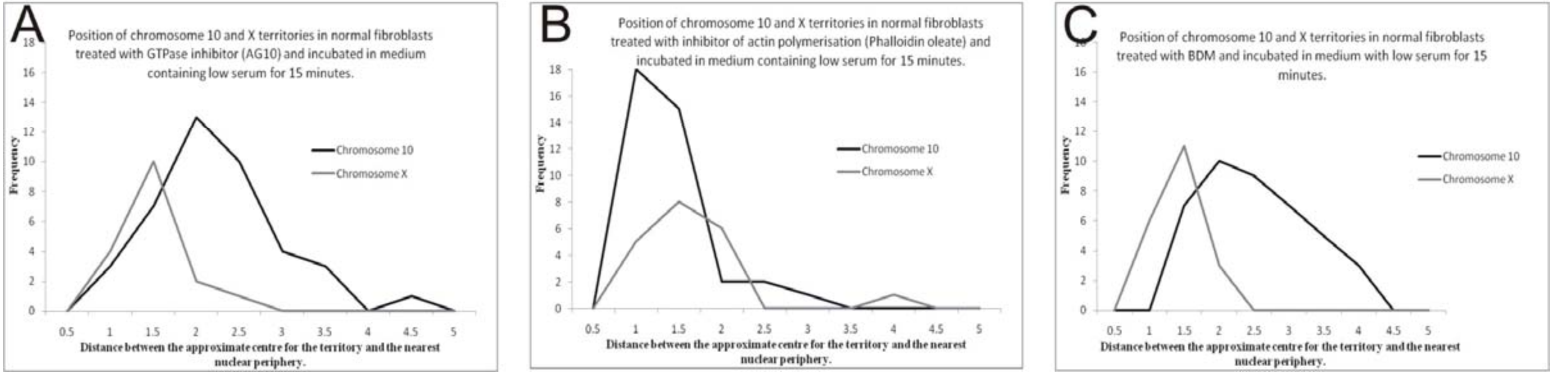

Figure 3: Nuclear locations of chromosomes 10 and X territories in normal 2DD cells treated with inhibitors of GTPase, actin and myosin polymerisation were confirmed using 3D-FISH analysis. The frequency distribution curves display the location of chromosome 10 and X territories in cells treated with GTPase inhibitor (A), in cells where actin polymerisation is inhibited using Phalloidin oleate (B) and in cells where nuclear myosin polymerisation is inhibited using BDM (C). Unpaired, unequal variance, two-tailed student's t-test at 95% confidence interval ($p<0.05$) has shown that there is no significant difference between the position of chromosome 10 in normal proliferating fibroblasts and fibroblasts treated with AG10 and BDM followed by incubation in low serum for 15 minutes. In cells treated with Phalloidin oleate and low serum for 15 minutes, the position of chromosome 10 is significantly different to its position in normal proliferating fibroblasts. While the position of chromosome X in cells subjected to any of these conditions do not show any significant difference to their normal proliferating counterparts.
