# Supplementary material for: Rapid chromosome territory relocation by nuclear motor activity in response to serum removal in primary human fibroblasts
Source: Genome Biol. 2010 Jan 13;11(1):R5. doi: 10.1186/gb-2010-11-1-r5 (PMC2847717; doi:10.1186/gb-2010-11-1-r5)
Supplement: Additional data file 4 — The DAPI distribution with each shell of the 2D erosion analysis script for each experiment performed, revealing that the DNA content did not alter after any of the treatments (4). [file gb-2010-11-1-r5-S4.pdf]

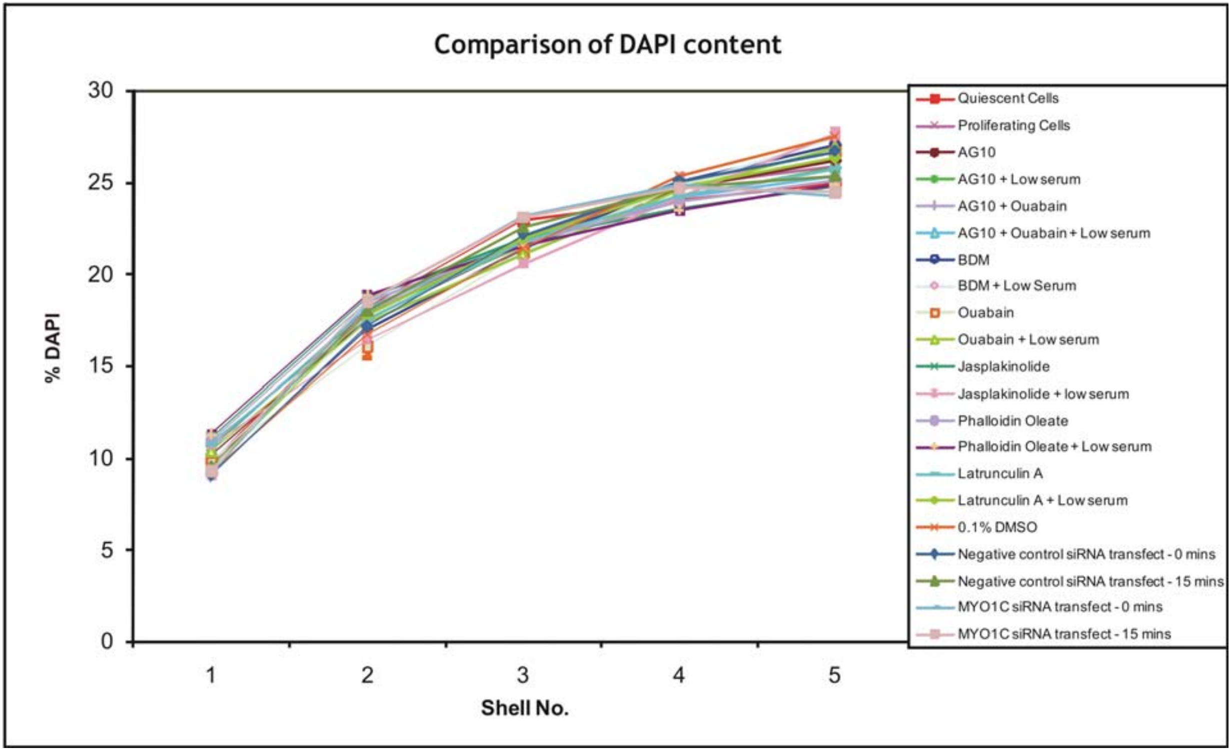

Figure 4: A line graph comparing the % of DAPI in each shell created by the erosion script for fibroblast nuclei under different situations analysed in this study.
