# Supplementary material for: Rapid chromosome territory relocation by nuclear motor activity in response to serum removal in primary human fibroblasts
Source: Genome Biol. 2010 Jan 13;11(1):R5. doi: 10.1186/gb-2010-11-1-r5 (PMC2847717; doi:10.1186/gb-2010-11-1-r5)
Supplement: Additional data file 5 — A table describing the inhibitors and drugs used in this study. [file gb-2010-11-1-r5-S5.docx]

Additional File 5 Table 1

**Table S1** The inhibitors and drugs used in this study. This table summarises the inhibitors used in the study, their respective substrates, concentrations that they were used at, the time duration they were used for and the reference from where this information was derived.

| **Inhibitor** | **Substrate** | **Final Concentration** | **Duration** | **References** |
| --- | --- | --- | --- | --- |
| BDM (Calbiochem) diluted in DMSO | Myosin | 10 mM | 15 minutes | [65-67] |
| Jasplakinolide (Calbiochem) diluted in DMSO | Myosin | 1 μM | 1 hour | [68] |
| Latrunculin A (Calbiochem) diluted in DMSO | Actin | 1 μM | 30 minutes | [62] [63] |
| Phalloidin Oleate (Calbiochem) diluted in water | Actin | 1 μM | 30 minutes | [64] |
| Ouabain (Calbiochem)  diluted in water | ATPase | 100 μM | 30 minutes | [59] |
| AG10 (Calbiochem)  diluted in DMSO | GTPase | 100 μM | 20 minutes | [60] [61] |
| Ouabain + AG10 (Calbiochem) | ATPase + GTPase | 100 μM of each | 30 minutes | [59, 60] [61] |
